# Confocal Raman microspectroscopy for spatially resolved tissue characterisation of disease‐linked spectra‐pathological signatures

**DOI:** 10.1002/ctm2.70487

**Published:** 2025-09-25

**Authors:** Nektarios A. Valous, Inka Zörnig, Dirk Jäger

**Affiliations:** ^1^ Applied Tumor Immunity Clinical Cooperation Unit National Center for Tumor Diseases German Cancer Research Center (DKFZ) Heidelberg Germany; ^2^ Medical Faculty Heidelberg Heidelberg University Department of Medical Oncology Heidelberg University Hospital (UKHD) Heidelberg Germany; ^3^ Center for Quantitative Analysis of Molecular and Cellular Biosystems (Bioquant) Heidelberg University Heidelberg Germany; ^4^ Department of Medical Oncology National Center for Tumor Diseases Heidelberg University Hospital (UKHD) Heidelberg Germany

**Keywords:** biochemical imaging, biophotonics, Raman microscopy, spectral analysis, tissue‐level insights

## Abstract

**Abstract:**

Raman spectroscopy is a versatile analytical technique for highly specific molecular characterisation of cells, biofluids and tissues. Confocal Raman microspectroscopy combines optical microscopy with Raman spectroscopy to spatially resolve biochemical changes in tissue samples. This work focuses on research articles that utilise confocal Raman microspectroscopy in human or murine tissue sections for identifying disease‐linked spectra‐pathological features. For scientists and clinicians who seek ideas in incorporating confocal Raman microspectroscopy into their experimental workflows, this piece provides a curated selection of studies (spanning cancer and cardiovascular diseases) that highlight key spectroscopic and biomedical insights. The lack of standardisation and the fragmentation of research protocols are major challenges that limit study reproducibility and prevent systematic cross‐validation. Moving forward, confocal Raman microspectroscopy, coupled with robust computational approaches, will continue to detect disease‐specific spatiotemporal biomolecular signatures, and integration with complementary imaging or omics methods will keep enhancing its ability to analyse complex biological systems and uncover disease progression mechanisms.

**Key points:**

- Confocal Raman microspectroscopy enables spatially resolved tissue characterisation and reveals disease‐linked spectra‐pathological features.
- Key challenges limit clinical translation, for example, lack of standardisation and insufficient reference spectral databases.
- Future research progress depends on interdisciplinary collaboration, robust computational methods and integration with complementary imaging or omics technologies for enhanced disease characterisation.

## Graphical abstract

Confocal Raman microspectroscopy for spatially resolved tissue analysis: technology setup, spectra‐pathological signatures derived from computational methods, and tissue‐level insights for biomedical research and clinical applications.

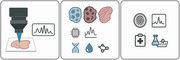

## 1 INTRODUCTION

Raman spectroscopy probes molecular vibrations (associated with chemical bonds) to obtain information on molecular structure, composition and intermolecular interactions.^1^ The vibrational frequencies (frequency differences between incident and scattered light; Raman shifts measured in wavenumbers: cm^−1^) at which Raman bands occur are characteristic of vibrational modes of specific bond types in molecules, with the intensity directly proportional to the concentration of molecular constituents that give rise to the bands.^1^ The spectroscopic profile arising from the Raman‐active functional groups of nucleic acids, proteins, lipids and carbohydrates can provide information on the biological components of cellular, biofluid and tissue samples.^1^, ^2^ Raman spectra are typically presented with the Raman shift (cm^−1^) on the *x*‐axis and the intensity (often in arbitrary units) on the *y*‐axis. By providing information on multiple molecules, the technique can generate a biochemical fingerprint (distinct and specific spectrum that allows the identification of a molecular compound) which may indicate the presence or absence of disease, and even the stage of disease progression.^3^, ^4^

Raman spectroscopy, in combination with chemometric data analysis, is a powerful and accurate technique for detecting pre‐cancerous and cancerous biochemical changes both in vitro and in vivo, for a range of malignant conditions.^5^ For example, the technique has been utilised to distinguish between normal, benign and malignant breast tissue based on specific spectral signatures.^2^ Raman spectroscopy has also great potential for the discovery of new biomarkers that can assist the clinical screening of disease susceptibility and determination of incidence rate.^6^ Compared with conventional chemical analytical techniques, Raman spectroscopy has the advantages of being non‐destructive, fast, highly repeatable, environmentally friendly, with minimal to no sample preparation, and capable of working on small samples.^6^ For complex samples (e.g., cells, biofluids, tissues), the observed Raman spectra are a superposition of all the spectra of the individual biochemical components, and the task is to decode the complex spectral signatures.^4^ Throughout this work, amino‐acid‐related bands should be interpreted as composite signals; in complex tissues, spontaneous Raman generally does not distinguish free amino acids from those incorporated into proteins by band position or intensity alone, therefore assignments reflect dominant contributors under the specific experimental context, unless selective measurements are provided. As a reminder, the Raman spectra of biological samples are divided into three spectral regions.^7^ The fingerprint region covers the range between 600 and 1800 cm^−1^; contributing bands to this region usually involve somewhat larger atoms (e.g., carbon, nitrogen, oxygen) or complexes of several hydrogen atoms.^7^ This region is rich in biochemical information including signatures associated with proteins, lipids and DNA.^8^ The high wavenumber region covers the range between 2500 and 3800 cm^−1^, corresponding to the molecular vibrations of lipids, proteins and water.^8^ Most bonds involving independently vibrating hydrogen atoms vibrate with much higher energies than other bonds, and these higher energy vibrations occupy this region.^7^ Both fingerprint and high wavenumber regions offer a more comprehensive biochemical overview of the sample.^8^, ^9^ The spectral region between 1800 and 2500 cm^−1^ (silent region) is mostly empty of contributions from biological molecules, although there are some exceptions (e.g., alkynes).^7^

Table 1 presents an overview of the major Raman scattering techniques: spontaneous Raman scattering, stimulated Raman scattering (SRS), coherent anti‐Stokes Raman scattering (CARS), surface‐enhanced Raman scattering (SERS) and tip‐enhanced Raman scattering (TERS).^10^ The spontaneous (typical examples are SERS and TERS, which can also utilise coherent Raman scattering) and coherent Raman scattering (e.g., SRS and CARS) processes are explained in Ref.,^11^ where the authors provide a tutorial‐style introduction to the fundamental physics at work. Table 2 presents a non‐exhaustive overview of the computational methods employed for analysing Raman data.^12^, ^13^

**TABLE 1:** Overview of major Raman scattering techniques.^10^

**Spontaneous Raman scattering** ^10^ Inelastic (Stokes and anti‐Stokes) scattering of light by molecules resulting in energy shifts that correspond to molecular vibrational states; weak effect (one in 10^8^ photons scattered). *Application scenario*: label‐free imaging of biological samples.
**Stimulated Raman scattering (SRS)** ^10^ Coherent process where pump and Stokes beams interact to enhance Raman signals; stronger signal than spontaneous Raman scattering with directional and coherent output. *Application scenario*: high‐speed imaging of biomolecules. *Recent advances*: SRS flow cytometry allowing high‐throughput single‐cell analysis without fluorescent labels.
**Coherent anti‐Stokes Raman scattering (CARS)** ^10^ Four‐wave mixing process involving pump, Stokes and probe beams to generate anti‐Stokes signals; high signal intensity (up to 10^6^× stronger than spontaneous Raman) but non‐resonant background causes spectra distortions. *Application scenario*: label‐free live‐cell imaging. *Recent advances*: dual‐wavelength CARS allows simultaneous imaging of multiple vibrational modes, and electro‐CARS allows the suppression of non‐resonant background signals.
**Surface‐enhanced Raman scattering (SERS)** ^10^ Enhancement of Raman signals by plasmonic nanostructures (e.g., gold or silver nanoparticles); signal enhancement up to 10^11^ due to localised surface plasmon resonances. *Application scenario*: trace detection of DNA and pathogens.
**Tip‐enhanced Raman scattering (TERS)** ^10^ Combination of Raman spectroscopy with scanning probe microscopy to achieve nanoscale spatial resolution (10‒30 nm); enhancement factors for TERS (10^3^‒10^6^ relative to spontaneous Raman) are weaker than SERS. *Application scenario*: imaging of single DNA/RNA strands.

**TABLE 2:** Non‐exhaustive overview of Raman data analysis methodologies.^12^, ^13^

**Pre‐processing** ^12^ *Denoising* (kernel smoothing, Savitzky‒Golay differentiation), *baseline removal* (morphological weighted penalised least squares, standard normal variate, multiplicative scatter correction), *cosmic ray removal* (sharp/abnormal spike detection, image curvature correction, mapping‐based technique), *scaling methods* (normalisation by peak, auto‐scaling, row/column normalisation, mean‐centering).
**Pattern recognition** ^12^ *Unsupervised and supervised methods* (principal component analysis, independent component analysis, vertex component analysis, hierarchical cluster analysis, *k*‐means, *t*‐distributed stochastic neighbour embedding, partial least squares discriminant analysis, linear discriminant analysis, *k*‐nearest neighbour, support vector machines, neural networks), *quantification methods* (partial least squares regression, multivariate curve resolution).
**Validation** ^12^ *Cross‐validation* (leave‐one‐out, random subsets), *permutation tests*, *confusion matrices*, *receiver operator characteristic curves*.
**Deep learning** ^13^ *Pre‐processing* (spectral pre‐processing and non‐resonant background removal using convolutional neural networks, spectral denoising using the ResUNet architecture), *classification and regression* (1D convolutional neural network models for classification tasks, ResNet architectures applied in classification scenarios to mitigate vanishing gradient issues, component identification from Raman spectra, generative adversarial network‐based augmentation methods combined with convolutional neural networks for pathogen classification), *highlighting* (convolutional neural networks for highlighting important spectral regions by extracting Raman peaks and suppressing background features).

The following section provides a selection of key review papers that can equip scientists and clinicians with the background needed to develop a practical understanding of the subject, which will then allow them to delve into more specialised literature.

## 2 INFLUENTIAL WORKS IN RAMAN SPECTROSCOPY

The biomedical field has many useful review articles^14^, ^15^, ^16^, ^17^, ^18^, ^19^, ^20^, ^21^, ^22^, ^23^, ^24^, ^25^, ^26^, ^27^, ^28^ that focus on the governing fundamental principles, the different Raman techniques along with applications (in vitro, ex vivo, in vivo) in cells, biofluids and tissues, and the combination of Raman scattering with other modalities, for example, matrix‐assisted laser desorption/ionisation time‐of‐flight (MALDI‐TOF) and Raman imaging.^29^ As a side note, Raman spectroscopy has also became indispensable in advancing pharmaceutical processes, and in Ref.,^30^ the authors explored current trends in Raman‐based analytics and its value in modern pharmaceutical applications. The experimental and data analysis pipeline in a typical Raman experiment includes the following steps: (i) formulating a biomedical question, which dictates experimental design, (ii) acquiring and storing data that contain metadata (e.g., acquisition parameters) and spectral data (e.g., vibrational spectra), (iii) pre‐processing of the raw data (e.g., cleaning, normalising and reducing noise), (iv) analysing the data using multivariate statistical techniques and machine/deep learning algorithms, and (v) visualising the results to detect patterns or features leading to interpretation in the context of the aforementioned biomedical question.^31^ The workflow includes iterations of assessment and validation, which involve statistical testing to evaluate the predictive power and generalisability of the models; these iterations guide the selection of the most appropriate analysis methods.^31^ The insights gained are utilised to refine the experiments and help discover new information about the biological system.^31^ Furthermore, appropriate data analysis strategies and typical algorithms for addressing biomedically driven research questions can be the following: (i) exploratory data analysis (relationships between samples; no prior information is required, e.g., principal component analysis, self‐organising maps), (ii) sample classification (samples classified into different groups; supervised learning is employed, e.g., linear discriminant analysis, partial least squares discriminant analysis, support vector machines, neural networks), (iii) quantitative analysis (correlations with continuous variables; quantitative prediction of unseen samples to some continuous variable, e.g., partial least square regression, principal component regression, support vector regression), and (iv) explanatory analysis (differences in the chemical/physical state among samples; spectral changes as a function of predefined groups, e.g., many algorithms can be employed).^31^

In our assessment, three reviews are particularly noteworthy in terms of thoroughness and in‐depth examination of subject matter, that is, covering all relevant aspects and offering insightful and current perspectives. Shipp et al. wrote an excellent review article that offers a solid starting point for researchers interested in Raman spectroscopy for the life sciences, with accessible theoretical background and practical examples.^7^ The authors discussed: (i) spontaneous and coherent Raman scattering, (ii) molecular vibrations and light interactions (classical and quantum mechanical treatments), (iii) molecular origins of common and most dominant Raman bands encountered when measuring biological samples, (iv) techniques currently used in Raman spectroscopy (spontaneous Raman scattering, CARS, SRS, SERS, fibre optic probes, selective scanning Raman spectroscopy, spatially offset Raman spectroscopy and Raman‐based labelling techniques) including biological applications, (v) methodological approaches to classification and modelling, and (vi) future directions of Raman spectroscopy for the life sciences.^7^ As a side note and in the context of Raman‐based labelling techniques, Chen et al. presented a multiplexed Raman probe platform for high‐content, live‐cell profiling at the single‐cell level; their platform enabled the simultaneous measurement of 14 different cellular features (e.g., cell surface protein expression, endocytosis and multiple metabolic activities) in live single cells, surpassing the multiplexing limits of fluorescence‐based methods.^32^ Butler et al. wrote a protocol about Raman spectroscopy as a label‐free technique to analyse biological materials.^33^ The authors discussed: (i) principles of Raman spectroscopy and key spectral regions corresponding to biomolecules, (ii) different Raman techniques and their clinical potential, (iii) experimental design focusing on instrumentation, sample preparation and spectral acquisition as well as calibration and spectral quality optimisation, (iv) data analysis including pre‐processing, feature extraction and classification, while covering different software options, (v) materials and protocols for sample preparation, spectral acquisition and data analysis, including troubleshooting tips, and (vi) anticipated results, for example, examples of pre‐processed spectra, classification outcomes and visualisations, including the impact of parameter choices on data quality.^33^ Regarding sample preparation, mammalian tissues are prepared either as formalin‐fixed, paraffin‐embedded (FFPE) sections or as fresh/snap‐frozen specimens.^33^ For FFPE work, blocks are cooled for more than 10 min to harden the wax, trimmed, sectioned at 5–10 µm on a microtome, ribbons are floated at 40°C–44°C, mounted on Raman‐grade CaF_2_ slides, air‐dried for approximately 30 min to ensure adhesion, then de‐waxed by three 5‐min xylene immersions and cleared with 15‐min washes in 100%, 90% and 70% ethanol; slides are then stored dry at room temperature and remain usable for 1 year.^33^ For interpretation, it is important to note that formalin modifies proteins by cross‐linking (alters spectral peaks associated with proteins in the 1500–1700 cm^−1^ region) and paraffin contributes strong bands at 892, 1065, 1135, 1174, 1298, 1421, 1443 and 1464 cm^−1^; these can be removed either chemically or computationally, but interpreting the lipid features should be done with caution because fixation and de‐waxing have a marked effect on lipid content.^33^ Fresh or snap‐frozen tissues may overcome these drawbacks but are harder to section and must be handled to limit degradation, for example, snap‐freezing in liquid nitrogen/isopentane and either rapid analysis or frozen storage for up to 1 year with minimal loss of integrity.^33^ Furthermore, substrate choice is critical for low background, for example, CaF_2_ or quartz slides are preferred.^33^ Cutshaw et al. summarised recent findings in Raman spectroscopy focusing on the changes in the metabolome and proteome during disease progression and in response to therapy.^34^ The authors discussed: (i) multivariate analysis and machine learning approaches with applications in classification, denoising and deconvolution of Raman spectral data, (ii) label‐free Raman spectroscopy for metabolic profiling with applications in gastrointestinal, cardiac and neurodegenerative diseases, (iii) integration with multiomics techniques (metabolomics and transcriptomics) showing examples in cancer, bacterial resistance and yeast studies, (iv) design and utility of metal nanoparticles for in vivo biomarker detection with examples in cancer imaging, treatment response and immune cell tracking, (v) multimodal SERS integrated with optical (fluorescence, photoacoustic) and clinical (magnetic resonance imaging, positron emission tomography, computed tomography) imaging, including applications in preoperative planning, intraoperative guidance and postoperative margin assessment, (vi) SERS‐based high‐throughput proteomic assays for the ultrasensitive detection of protein biomarkers in cancer, Alzheimer's disease and cardiovascular disease, and (vii) metal‐free nanoprobes for the Raman silent region with applications in live‐cell imaging and in vivo studies.^34^

The following section outlines the principal focus of this work, which is the utilisation of confocal Raman microspectroscopy for obtaining tissue‐level biochemical insights.

## 3 PRINCIPAL FOCUS

Raman spectroscopy, in the biomedical domain, is a versatile analytical technique for highly specific molecular characterisation of cells, biofluids and tissues. Raman microscopy, integrating Raman spectroscopy with optical imaging, is a powerful technique that delivers comprehensive chemical information across spatial and spectral dimensions.^35^ Because of its high chemical specificity and minimal to no sample preparation requirements, Raman imaging has found broad adoption in biological sample analysis with a variety of applications.^35^ Confocal Raman microspectroscopy is one of the most commonly used Raman imaging techniques, in which the laser is focused to a small spot within the sample and the Raman scattered light is filtered by a pinhole to make sure the collected photons are from the focal plane,^36^, ^37^ visualised in Ref.^18^ This technique acquires a spectrum at each point on a sample plane, temporarily dwelling in position for data collection while scanning across the tissue to generate volumetric data (hyperspectral data cube).^27^ The key components of a confocal Raman microscope are the following: laser source (e.g., 532 and 785 nm), objective lens (typically with high numerical aperture), confocal pinhole (enabling optical sectioning), spectrograph (separating the collected Raman‐scattered light into its component wavelengths) and CCD detector (high sensitivity, low noise). The objective lens is an influential factor with respect to the lateral resolution (ability to distinguish two points separated in the *x‒y* plane) of a Raman microscope; the resolution depends on the numerical aperture of the objective which determines the tightness of the focus (most high‐end objectives have values ranging from 0.9 to 0.95).^38^ The size of the pinhole also plays a crucial role in the resolution and contrast of the Raman images; as the size of the pinhole decreases, light from out‐of‐focus regions is more effectively rejected by the system resulting in higher spatial resolution at the expense of low signal intensity that can reduce image contrast.^38^ Confocal Raman microspectroscopy (based on spontaneous Raman scattering) has been widely used in life science research for its ability to provide molecular fingerprint information with high spatial resolution.^39^ Even though the spontaneous Raman effect is inherently weak (due to the intrinsic low Raman scattering efficiency) with longer acquisition times, the imaging speed in spontaneous Raman microscopy systems has improved dramatically in the last several years.^40^ Recently, a variant was developed to achieve very fast Raman imaging; the authors developed a technique called multiline illumination confocal Raman microscopy, where the detection of separate sample regions is performed in parallel enabling spontaneous Raman imaging as fast as ∼10 min/megapixel, therefore expanding the applications of Raman microscopy in the biomedical field.^40^

In principle, providing ready‐to‐use systems with stable optics and integrated software enables scientists and clinicians to conduct imaging and characterisation studies focusing on scientific questions and data interpretation rather than dealing with instrument setup and troubleshooting. The following section provides a focused overview of research articles that utilise label‐free bioimaging of human or murine tissue sections by means of confocal Raman microspectroscopy for identifying disease‐linked spectra‐pathological features. For scientists and clinicians who seek ideas in incorporating confocal Raman microspectroscopy into their experimental workflows, this work provides a curated selection of studies (spanning cancer and cardiovascular diseases) that highlight key spectroscopic and biomedical insights; these studies (selected for their good experimental design and analysis workflows) capture the main lines of investigation currently reported for confocal Raman microspectroscopy on tissue sections in biomedical research. Unlike broad reviews that cover general advances in the field and often include long reference tables that list research papers, this work concentrates specifically on the use of confocal Raman microspectroscopy for the spatially resolved characterisation of tissue sections in different disease contexts. The selected research articles are chosen for their direct relevance to identify and interpret disease‐linked spectra‐pathological features; Raman biomarkers of thoracic aortic aneurysm lesions, multivariate staging and grading of breast cancer, discrimination of intraductal carcinoma of the prostate from other lesions, repurposing single‐cell/spatial omics analytics for label‐free Raman tissue profiling, and in situ mapping of the oncometabolite fumarate with subcellular compartmentalisation in cells and tissues. Each study is discussed with an emphasis on its biomedical significance, particularly as it pertains to linking spectroscopic findings to underlying molecular and pathological changes in tissues. By presenting relevant and impactful studies, the review aims to facilitate the effective use of this technique for tissue analysis and encourage further advances in disease‐related research.

**FIGURE 1:**
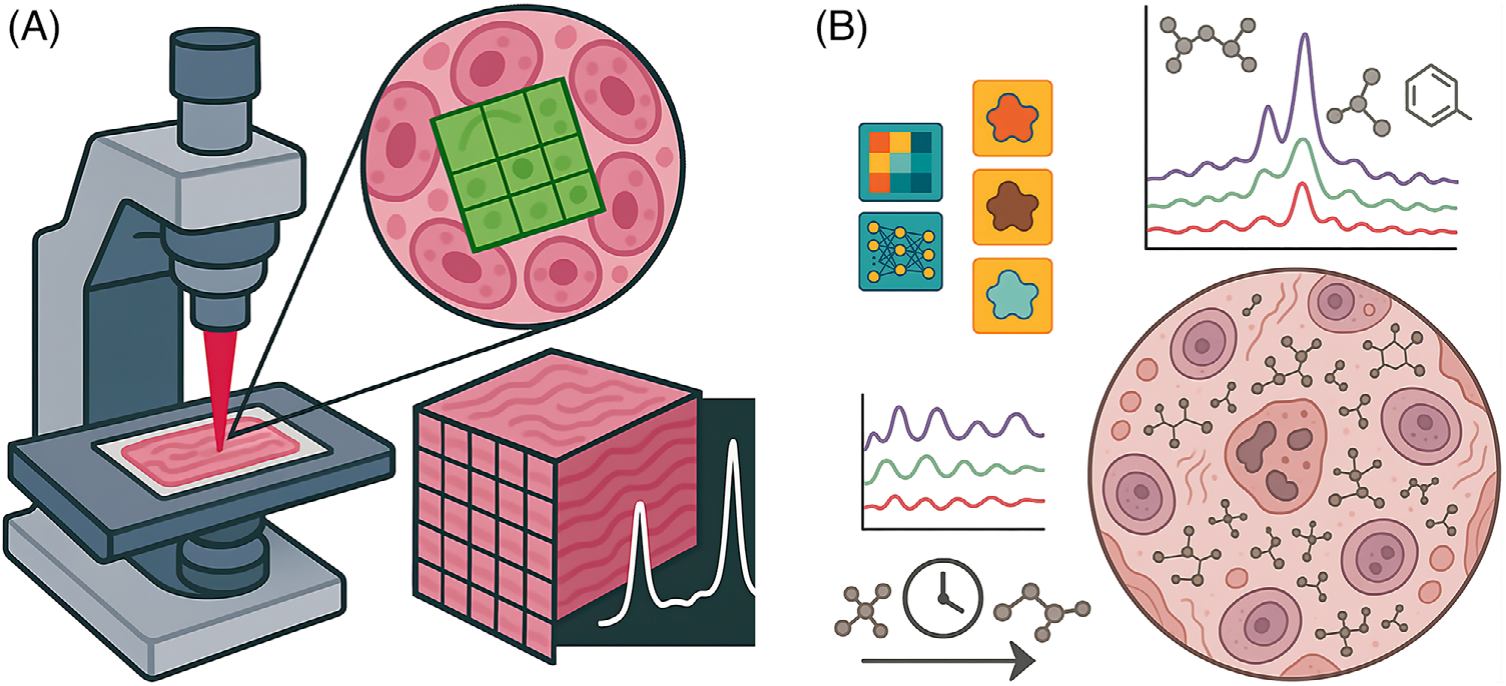
Confocal Raman microspectroscopy for biochemical tissue characterisation: (a) a confocal Raman microscope focuses a laser onto a tissue section enabling spatially resolved biochemical analysis, where a magnified region of interest shows a grid of pixels (each pixel corresponding to a single Raman spectrum) highlighting how the technique can generate hyperspectral data cubes that combine spatial and spectral information for localised biochemical profiling. (b) Raman spectra tissue profiles reflect the biomolecular variation across different regions or compartments, enabling the spatiotemporal detection of dynamic biochemical processes and the capturing of disease‐linked biochemical signatures at high spatial resolution; these insights are obtained through data analysis and visualisation utilising multivariate statistical techniques and machine/deep learning methods.

Table 3 outlines the general advantages and disadvantages of confocal Raman microspectroscopy. Figure 1 presents a general schematic of confocal Raman microspectroscopy for biochemical tissue characterisation showing both experimental setup and data acquisition as well as analytical and interpretative aspects.

**TABLE 3:** General advantages and disadvantages of confocal Raman microspectroscopy.

**Advantages**: Chemical specificity based on vibrational modes without the need of labels (external molecules or tags); high spatial resolution; optical sectioning and volumetric reconstruction for depth profiling and 3D imaging; sample remains practically intact; minimal to no sample preparation; compatibility with aqueous environments unlike infrared spectroscopy.
**Disadvantages**: Weak signal intensity leads to longer acquisition times; fluorescence (under visible laser excitation) may mask the Raman signal; less effective for detecting trace analytes due to weak signal and spectral overlap; complex and costly instrumentation requiring, for example, precise optics and sensitive detectors; high‐resolution spatial mapping can be slow due to point‐by‐point data acquisition.

## 4 SELECTED APPLICATIONS TO HUMAN DISEASE

### 4.1 Spectral biomarkers specific to thoracic aortic aneurysms

**FIGURE 2:**
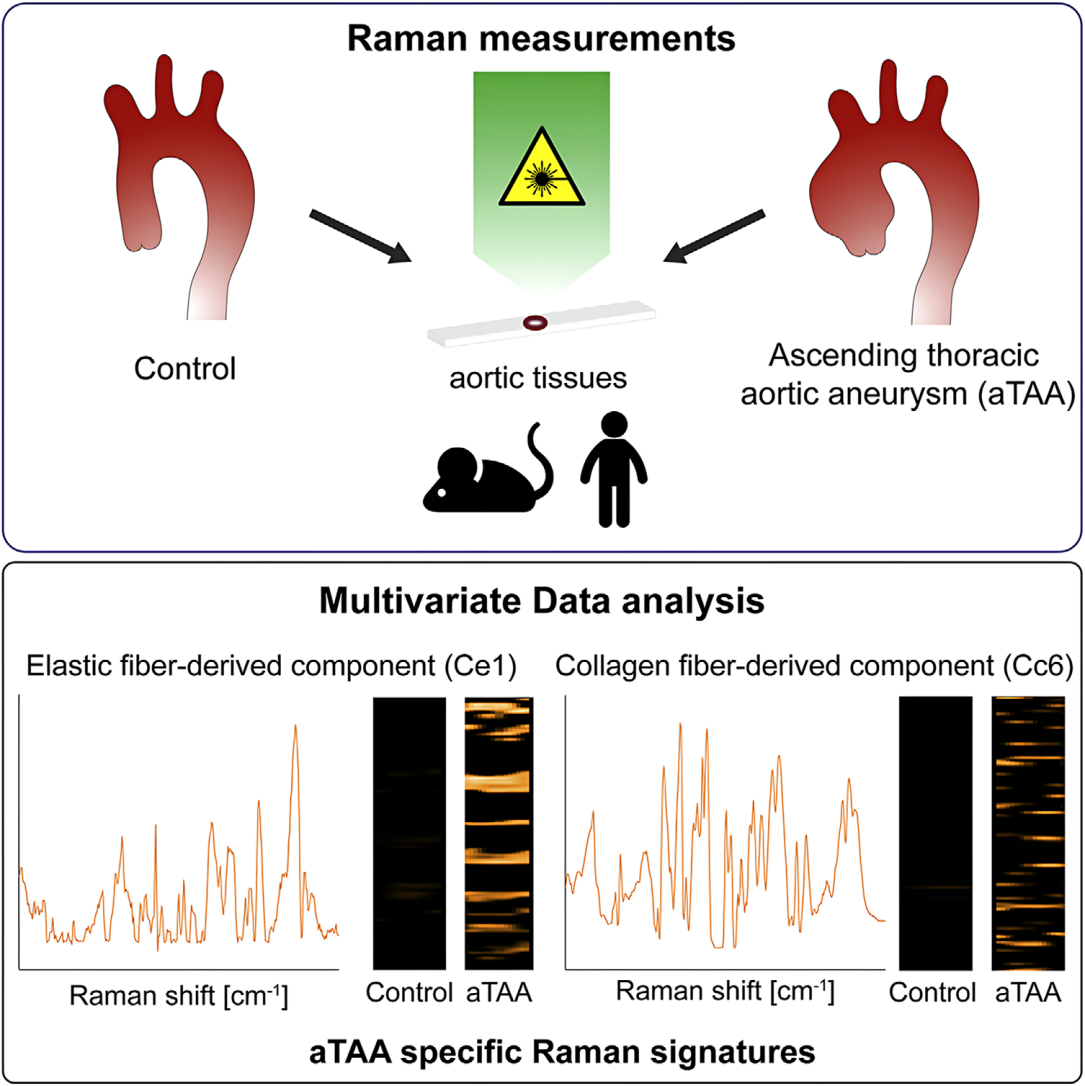
Raman imaging of human and murine tissue. Sugiyama et al. performed label‐free Raman imaging of human and murine ascending thoracic aortic aneurysms (aTAAs); using Raman spectra and multivariate data analysis, the authors identified spectral biomarkers for aTAA in elastic fibres and collagen fibres (Ce1 and Cc6, respectively) that are significantly increased in aTAA lesions.^41^ Figure reprinted from Ref.^41^ under the Creative Commons CC‐BY license, © 2021 The Authors.

Sugiyama et al. utilised confocal Raman microspectroscopy and multivariate data analysis (true component analysis [TCA], principal component analysis [PCA] and multivariate curve resolution [MCR]) to identify spectral biomarkers specific for ascending thoracic aortic aneurysms (aTAAs) in murine (genotypes: *Fbln4^SMKO^*, *Fbln5^KO^* and wild‐type [WT]) and human tissue.^41^ PCA identified differences in spectral data between WT, *Fbln5^KO^* and *Fbln4^SMKO^* tissues.^41^ MCR decomposed Raman spectra into subcomponents (Ce1‒Ce16 for elastin and Cc1‒Cc12 for collagen).^41^ Immunofluorescence (IF) staining (collagen type I, aggrecan, versican and nuclei) and histological analyses (alcian blue and oil red O visualising glycosaminoglycans and lipids, respectively) were also performed to compare and evaluate the performance of Raman imaging.^41^ Five major spectral components were identified, which were assigned to the Raman signatures of elastic fibres, collagen fibres, nuclei, lipids and residual extracellular matrix.^41^ Elastic fibres showed relevant bands at 528, 957 and 1108 cm^−1^ for desmosine and isodesmosine.^41^ Collagen fibres had Raman bands at 817 cm^−1^ (C‒C stretch) as well as 855, 878, 921 and 938 cm^−1^ for proline and hydroxyproline, and 1670 cm^−1^ for amide I.^41^ The identified spectra were used as reference spectra for subsequent TCAs.^41^ In the Raman imaging experiments, the authors showed that WT aortas contained solid native elastic fibres, whereas *Fbln5^KO^* elastic fibres showed disruptions.^41^ Collagen fibres were expanded from the adventitia to the medial layers in the *Fbln4^SMKO^* aortas, and aggrecan was markedly accumulated in *Fbln4^SMKO^* aortas.^41^ In the IF experiments, the *Fbln4^SMKO^* aortas showed strong signals in the intima and medial layers, while Alcian blue staining exhibited a massive accumulation of proteoglycans in *Fbln5^KO^* and *Fbln4^SMKO^* but not in WT.^41^ From the PCA analysis, the scores plot of PC‐4 against PC‐5 showed a clustering of all genotypes; PC‐4 showed a significant difference between WT and *Fbln4^SMKO^* as well as *Fbln5^KO^*.^41^ The corresponding molecular differences showed collagen‐related peaks for proline and hydroxyproline (862 and 944 cm^−1^) and amide III (1670 cm^−1^) bands.^41^ Elastic fibres in *Fbln5^KO^* and *Fbln4^SMKO^* appeared similarly disrupted at the microscopic level; however, Raman imaging and PCA separated the genotypes.^41^ From the MCR analysis, Ce1 was significantly different in *Fbln4^SMKO^* compared to WT and *Fbln5^KO^*; Ce1 was assigned to a non‐elastin‐related substructure of the elastic fibre.^41^ Cc6 was only detectable in *Fbln4^SMKO^* indicating abnormal molecular modification; Cc6 spectrum contained amino acid residues, including phenylalanine, tyrosine, tryptophan, cysteine, aspartic and glutamic acid.^41^ Regarding the human aTAA samples, Ce1 in aTAA samples was significantly different from control, and Cc6 increased in the human aneurysms (similar to the murine aneurysms).^41^ MCR further decomposed elastic fibre and collagen fibre components and identified aneurysm‐specific molecular signatures in human aTAA; Ce1 derived from abnormal proteins associated with aneurysmal lesions (detectable only in aTAA and *Fbln4^SMKO^*) and the spectrum of Cc6 (a protein‐like component) featured high intensities of several amino acid residues, indicating compositional changes of collagen molecules.^41^ Effectively, Ce1 and Cc6 have high potential as diagnostic biomarkers for aortic aneurysms.^41^ This study is a fine example of highlighting the sensitivity of Raman microspectroscopy for the detection of lesion‐specific biochemical differences (Figure 2).^41^

### 4.2 Tumour, node, metastasis staging and grading of breast cancer

**FIGURE 3:**
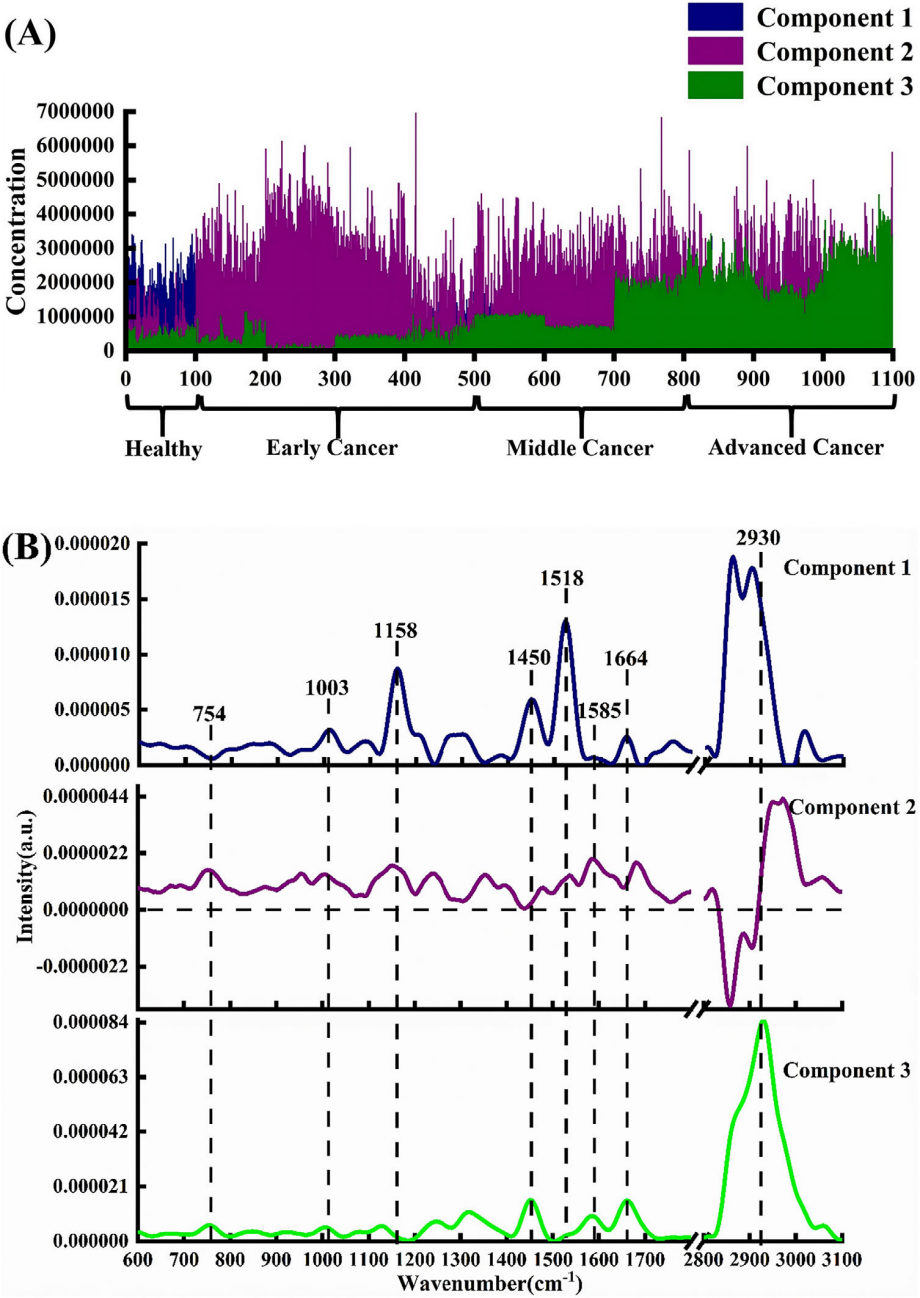
Concentration distribution characteristics of healthy group, and early/middle/advanced cancer acquired by multivariate curve resolution alternating least squares (MCR‐ALS): (a) spectral components of the concentration distribution characteristics (horizontal axis representing the number of spectral points), and (b) specific attribution of representative biochemical components corresponding to the concentration profile feature.^42^ Figure reprinted from Ref.^42^ with permission from Elsevier B.V., © 2022 Elsevier B.V.

Zhang et al. conducted a Raman‐based breast cancer evaluation based on molecular and functional changes during tumour progression (which produce distinct biomolecular fingerprints) thus bridging Raman spectroscopy with the tumour, node, metastasis (TNM) system.^42^ The samples included healthy, ductal carcinoma in situ (stage 0), and invasive ductal carcinoma tissue sections (IDC, stages I, II and III; grades 1–3 each).^42^ The main spectral characteristic peaks could be observed at 754 cm^−1^ (tryptophan), 1003 cm^−1^ (phenylalanine), 1158 cm^−1^ (β‐carotene), 1450 cm^−1^ (lipids), 1518 cm^−1^ (β‐carotene), 1585 cm^−1^ (nucleic acids), 1664 cm^−1^ (proteins and fatty acids) and 2930 cm^−1^ (saturated bonds of lipids, fatty acids and polypeptide proteins).^42^ The authors found that the spectral intensities of tryptophan, nucleic acids and proteins were higher in the cancer groups while the spectral intensities of phenylalanine, β‐carotene and lipids were higher in the healthy group.^42^ More specifically, when breast tissue transformed from healthy to advanced cancer, the normalised peak intensity of tryptophan (at 754 cm^−1^) displayed an increasing trend between healthy and cancer samples and then slowly decreased after tumour progression, which may be linked to the modulation of tumour cell proliferation.^42^ The phenylalanine peak (at 1003 cm^−1^) showed a similar changing pattern as the tryptophan peak; however, the intensity of the decreasing trend was more pronounced during tumour progression.^42^ Both β‐carotene peaks (at 1158 and 1518 cm^−1^) shared a uniform decline in intensity possibly attributed to the free radical oxidation of carotenoids.^42^ In the early cancer group, the intensity variations of proteins and fatty acids at 1664 and 2930 cm^−1^ were significantly lower than those found in healthy tissue.^42^ The decreased content of lipids in the cancerous group compared to the healthy group may be related to lipofuscin and lipid peroxidation.^42^ Conversely, the intensity of nucleic acids (at 1585 cm^−1^) in early cancer was significantly higher than in healthy tissue.^42^ MCR alternating least squares (MCR‐ALS) was applied to interpret the biochemical composition variations of breast cancer from healthy group to advanced cancer (Figure 3).^42^ Phenylalanine (1003 cm^−1^) and β‐carotene (1158 and 1518 cm^−1^), represented by component 1, were found in the healthy and early cancer groups.^42^ Component 3 became more pronounced in mid‐stage and advanced cancer comprising tryptophan (754 cm^−1^), lipids (1450 cm^−1^), nucleic acids (1585 cm^−1^) and proteins and fatty acids (1664 cm^−1^).^42^ In order to obtain a reliable spectral correlation with pathological processes (single peak analysis was insufficient for staging/grading due to overlapping spectral features), generalised discriminant analysis was performed for identifying the staging and grading status of breast cancer.^42^ The integrated areas under the receiver operator characteristic curves were 0.976, 0.947, and 0.991 for the multiclass discrimination of early‐stage, mid‐stage and advanced cancer, respectively.^42^ In Raman imaging experiments, *k*‐means cluster analysis visualised ductal structure evolution: tumour cells infiltrated and destroyed the ductal structure as cancer progressed.^42^ In tumour cell clusters, spectral intensity was the highest at 754, 1585 and 1664 cm^−1^ (tryptophan and nucleic acids), while in the peri‐cancerous areas, peaks at 1003, 1158 and 1518 cm^−1^ were higher (indicating that β‐carotene was concentrated in these regions).^42^ This study demonstrated the usefulness of confocal Raman microspectroscopy for the identification of biochemical changes that occur during breast cancer progression, and in linking these changes to the TNM staging and grading status.^42^

### 4.3 Prostate cancer and intraductal carcinoma of the prostate: multicohort study

Grosset et al. conducted a confocal Raman microspectroscopy study utilising tissue microarray sections from three multi‐institutional cohorts (CHUM, UHN and CHUQc‐UL) comprising 483 patients aimed at differentiating prostate cancer (PC) from benign prostate epithelium, as well as differentiating intraductal carcinoma of the prostate (IDC‐P) from PC and benign prostatic epithelium, including high‐grade prostatic intraepithelial neoplasia (HGPIN).^43^ A precise diagnosis of IDC‐P is a challenge for pathologists, since no specific biomarker is clinically available to reliably identify this aggressive histological variant of PC.^43^ The authors showed that Raman spectroscopy combined with machine learning could be utilised as a specific molecular biomarker of IDC‐P.^43^ Machine learning models were trained using the CHUM cohort and validated on the UHN and CHUQc‐UL cohorts to avoid data leakage and improve clinical robustness.^43^ Four models were developed: (i) identification of lymphocyte clusters within the prostate tissue, (ii) benign versus malignant prostate epithelial cells, (iii) IDC‐P versus invasive carcinoma, and (iv) HGPIN versus IDC‐P.^43^ The first one served as a performance benchmark due to the clear histological distinction (lymphocytes vs. PC cells; accuracy: 98%, sensitivity: 99%, specificity: 98%) with similar performance on the UHN and CHUQc‐UL cohorts.^43^ For the second one, the results for the UHN and CHUQc‐UL cohorts were, respectively, accuracy: 84%, sensitivity: 84%, specificity: 82%, and accuracy: 86%, sensitivity: 87%, specificity: 81%.^43^ Thirty‐two important Raman spectral differences were identified with 10 Raman peaks contributing the most to the classification.^43^ The peaks at 1450 and 1484 cm^−1^ were significantly increased in PC compared to benign tissue; biochemical constituents assigned to these peaks were mostly from DNA and RNA, and the backbone of protein and lipids.^43^ PC tissue also showed reduced levels of several amino acids (proline, tyrosine, valine, phenylalanine, tryptophan).^43^ The results confirmed the diagnostic potential of Raman microspectroscopy for detecting PC, suggesting molecular distinctions between benign and cancerous prostate epithelial cells, particularly with regard to nucleic acid and protein composition.^43^ For the third one, the results for the UHN and CHUQc‐UL cohorts were, respectively, accuracy: 91%, sensitivity: 88%, specificity: 93%, and accuracy: 85%, sensitivity: 85%, specificity: 86%.^43^ IDC‐P showed increased DNA and RNA backbone and protein secondary structures (α‐helix and β‐sheet, specifically for the amide III peak). Tyrosine, proline, valine and phenylalanine were also elevated in IDC‐P, while guanine, tryptophan, fatty acids and the amide I peak from the protein α‐helix were decreased.^43^ From the Raman peaks that were associated with IDC‐P, two were previously shown to be associated with end‐stage castration‐resistant PC (1171 and 1247 cm^−1^).^43^ As the association between IDC‐P and castration‐resistant PC is well established, the results support the value of the classification models.^43^ For the fourth and final one, IDC‐P could be distinguished from HGPIN with accuracy >97%.^43^ Five major peaks contributed the most; HGPIN had higher levels of adenine while IDC‐P had increased phenylalanine (1003 cm^−1^) and the β‐sheet secondary structure (1242 cm^−1^).^43^ The distinction between HGPIN and IDC‐P could be critical in refining diagnosis and treatment stratification.^43^ In this study, the authors utilised three independent non‐overlapping cohorts and compared to earlier studies, this design greatly enhanced generalisability and statistical power.^43^

### 4.4 Heterogeneity and dynamics within diseased cardiac tissues

**FIGURE 4:**
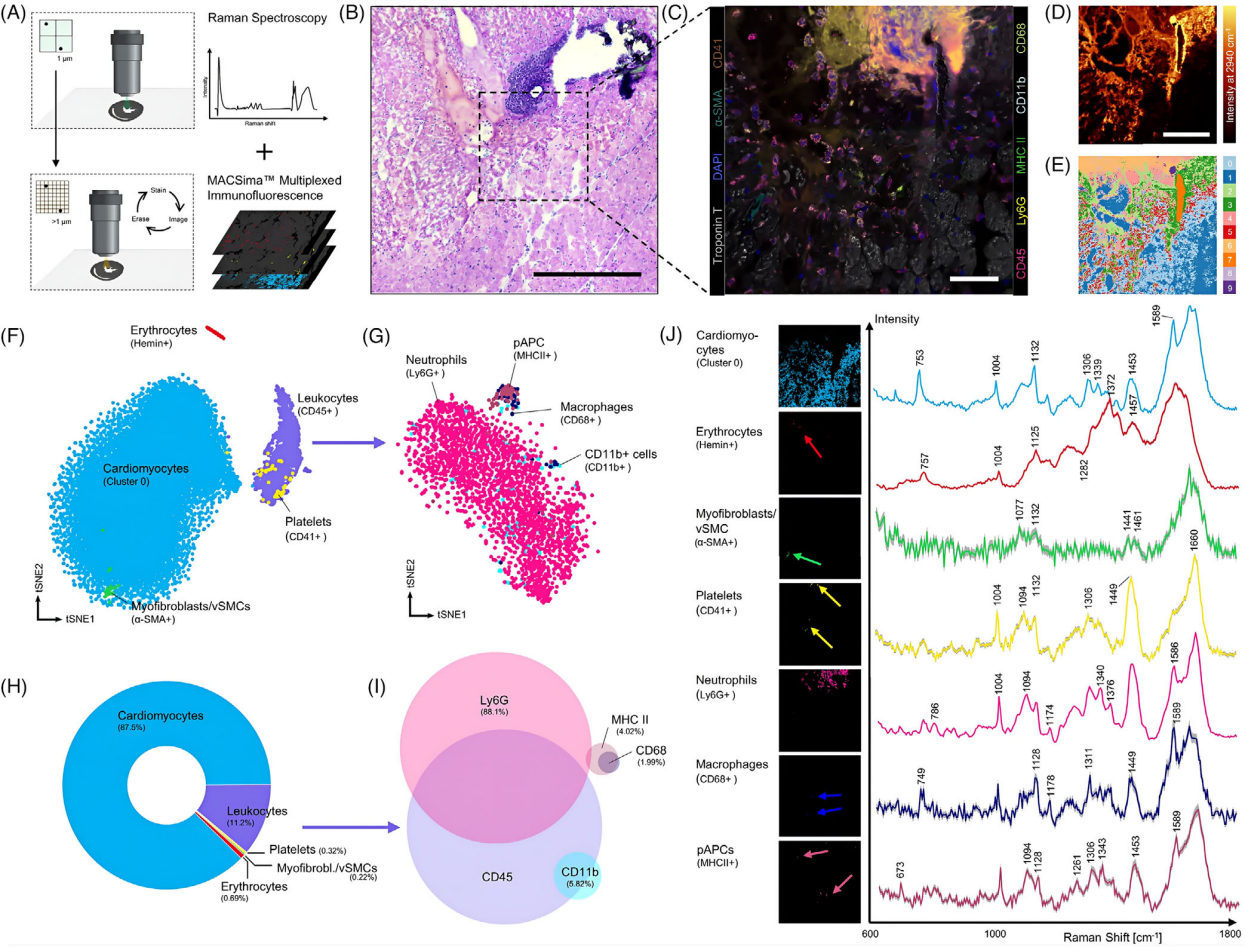
Defining the surrounding cellular (immune‐) landscape in the model of acute myocardial infarction: (a) multi‐omics approach to decipher spectra from cells by combination of Raman spectroscopy with consecutive multicolour immunofluorescence staining; (b) haematoxylin and eosin staining of an adjacent section of murine myocardial infarction (scale bar: 300 µm); (c) multiplexed immunofluorescence imaging was performed on the identical section and region as Raman spectroscopy was done previously (scale bar: 50 µm and *n* = 1 individual sample); (d) Raman intensity images at 2940 cm^−1^, which is the general band for lipids and proteins (scale bar: 100 µm); (e) cluster image identified by the Seurat workflow with cluster 0 being assigned to myocardium; (f) t‐SNE (t‐distributed stochastic neighbor embedding) plot after dimensionality reduction of Raman spectra of identified cell types showing clear separation into cardiomyocytes, erythrocytes and immune cells; (g) spectral subphenotyping of immune cells with neutrophils displaying the largest cluster, while MHC II+ professional antigen‐presenting cells (pAPCs) and CD68+ macrophages separating into a distinct cluster; (h) donut chart showing frequency distribution of identified cells (percent from absolute number of analysed number of pixels); (i) Venn diagram of surface markers analysed for the immune cell subpopulation, for example, most Ly6G+ cells were also CD45+; and (j) average spectra for the different cell types and characteristic peaks, together with the spatial representation of the analysed pixels (vascular smooth muscle cells [vSMCs]).^44^ Figure reprinted from Ref.^44^ under the Creative Commons CC BY 4.0 license, © 2023 The Authors.

Sigle et al. employed spatially resolved Raman microspectroscopy to obtain biomolecular information on diseased cardiac tissues by combining the technique with spatial metabolomics for the multiomics analysis of infarcted tissue, and by extending it with multiplexed IF for exploring the immune cell landscape of myocardial infarction.^44^ The authors employed two widely‐used (in cardiovascular research) mouse models: acute injury (myocardial ischaemia/reperfusion injury by transient ligation of the left anterior descending artery) and chronic injury (continuous infusion of angiotensin II in atherosclerosis‐prone apolipoprotein E‐deficient mice leading to cardiac hypertrophy and fibrosis).^44^ Spatial transcriptomic bioinformatics tools like Seurat (spatially unaware cluster analysis), BayesSpace (spatially aware clustering) and Monocle (pseudotime analysis) were repurposed for the analysis of Raman data.^44^ The unsupervised clustering of Raman spectra showed distinct segregation of pixels into biologically relevant clusters.^44^ Clusters 0 and 1 were enriched in assignments to peaks typically found in the myocardium: 1005, 1457 and 1640–1668 cm^−1^.^44^ Cluster 4 was identified as the collagen signature showing characteristic peaks at 858, 940, 1249 and 1680 cm^−1^.^44^ Cluster 6 showed peaks at 784, 1096, 1376 and 1580 cm^−1^ often reported for nucleic acids.^44^ Cluster 7 demonstrated the overall strongest peak intensities especially between 1200 and 1700 cm^−1^.^44^ PCA between myocardial subclusters identified differences in peaks at 758 cm^−1^ (tryptophan), 823 and 853 cm^−1^ (tyrosine), 1005 cm^−1^ (phenylalanine), and the amide I band between 1605 and 1650 cm^−1^, suggesting differences in protein composition and metabolism during cardiac remodelling.^44^ Essentially, cluster 1 (remodelled myocardium) represented a transition zone between the healthy myocardium (cluster 0) and fibrosis (cluster 4), with cluster 3 representing a pre‐fibrotic boundary around the actual fibrosis.^44^ Pseudotime trajectories uncovered a highly branched track in the region of the myocardial clusters; this result suggests high heterogeneity within the myocardial spectra.^44^ Spatial trajectories towards fibrosis revealed intensity shifts for hydroxyproline from collagen (858 cm^−1^) and cytochrome c in cardiomyocytes (1318 cm^−1^).^44^ The increase of the collagen band indicated a pre‐fibrotic remodelling of the myocardium close to the region of subendocardial fibrosis.^44^ Raman‐MALDI analysis demonstrated metabolic changes and also identified additional metabolites such as histidinyl‐serine and lysophosphatidylcholine.^44^ NADH was decreased at the infarct border and elevated in the ischemic region, suggesting a mitochondrial compensatory mechanism for energy delivery in the ischaemia/reperfusion region and marked tissue damage at the infarct border.^44^ In contrast, glucose was increased in the peri‐infarct area but decreased in adjacent areas.^44^ Raman spectroscopy also facilitated immune‐cell phenotyping through integration with multiplexed IF (Figure 4).^44^ Cell‐specific spectral signatures for erythrocytes, myofibroblasts/vascular smooth muscle cells, leukocytes, platelets, neutrophils, macrophages and antigen‐presenting cells were identified.^44^ In this context, the results suggested that Raman spectroscopy has high specificity but low sensitivity when detecting and delineating cell types.^44^ In summary, the authors presented a system‐level approach for the unbiased exploration of spatially resolved molecular patterns through Raman spectromics, utilising established bioinformatics tools and encouraging integration with other platforms for multimodal analysis.^44^

### 4.5 Spatial distribution of fumarate in cells and tissues

**FIGURE 5:**
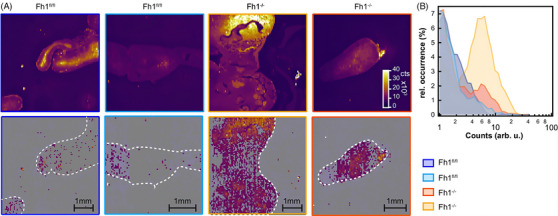
Fumarate mapping in tissue section: (a) Raman intensity maps for the 1130 cm^−1^ band used to show the outline of the mouse kidney tissue section (top row), and fumarate concentration maps obtained by fitting the 1401 cm^−1^ band (white outline traced from the Raman intensity maps serving as a guide to the eye, bottom row); (b) relative occurrence of fumarate concentrations in each area scan normalised by scan size: measured concentrations likely varied from true concentrations due to variations in focusing over the specimen; however, *Fh1^fl/fl^* tissues can be distinguished from induced *Fh1^−/−^* tissues by whether the majority of calculated concentrations are above the limit of detection.^46^ Figure reprinted from Ref.^46^ under the Creative Commons CC BY 4.0 license, © 2024 The Authors.

Fumarate is a key metabolite produced primarily by the tricarboxylic acid and urea cycles.^45^ New research has highlighted its involvement in immune regulation, revealing significant roles in both innate and adaptive immunity across autoimmune diseases, cancer and other conditions.^45^ The anti‐inflammatory effects of fumarate have been therapeutically exploited to treat autoimmune diseases; however, it has become clear that cancer and other immunosuppressive diseases may hijack this anti‐inflammatory property to suppress immune responses and avoid immune detection.^45^ Mutations in the gene encoding fumarate hydratase (FH) can lead to profound cellular metabolic alterations and fumarate accumulation, which can predispose to hereditary leiomyomatosis and renal cell cancer syndrome.^46^ FH loss or transcriptional downregulation is also well established in other cancers implying a key role in tumourigenesis.^46^ Kamp et al. demonstrated that confocal Raman microspectroscopy can detect fumarate in living cells in vivo and animal tissues ex vivo, and that it can distinguish between Fh1‐deficient and Fh1‐proficient cells based on fumarate concentration.^46^ For measuring metabolites in solution, powders were dissolved in deionised water to a concentration of 200 mM.^46^ Series of fumarate concentration dilutions were prepared from a 1 M stock solution.^46^ The fumarate powder spectrum was dominated by four peaks at 913 ± 2 cm^−1^, 1296 ± 2 cm^−1^, 1431 ± 2 cm^−1^ and 1657 ± 2 cm^−1^.^46^ In aqueous solution, fumarate displayed dominant peaks at 1277 ± 2 cm^−1^ (C‒H deformation mode), 1401 ± 2 cm^−1^ (fully symmetric CO_2_^−^ stretch), and 1652 ± 2 cm^−1^ (C = C stretch/CO_2_^−^ symmetric bending mode).^46^ For the fumarate detection in cell lines, two FH‐deficient cell lines (*Fh1^−/− Cl1^* and *Fh1^−/− Cl19^*) and their isogenic control (*Fh1^fl/fl^*) referred to respectively as knock‐out (Fh1‐KO) and wild‐type (Fh1‐WT) were utilised.^46^ For the cell measurements, the authors integrated a custom microscope chamber to maintain 37°C and 5% CO_2_ and mounted sub‐confluent cells on quartz coverslips to minimise background (<500 cm^−1^) and to permit a short‐working‐distance 60× water‐immersion objective.^46^ Coverslips were mounted in an Attofluor cell chamber with phenol red free liquid cell culture medium.^46^ Area scans at 532 nm (26 mW, 0.3 s dwell, 0.5 µm steps) covered a single cell in approximately 10 min.^46^ These practical choices (environmental control, low‐background quartz, water‐immersion optics and short dwell times) enabled the robust Raman mapping of intracellular fumarate.^46^ The fingerprint Raman spectral region of cells included the CH_2_ deformation (1445 cm^−1^), amide I (1656 cm^−1^) and amide III (1250 cm^−1^) bands, as well as DNA and cytochrome c bands.^46^ The 1401 cm^−1^ fumarate band was distinguishable, appearing stronger in Fh1‐KO cells compared to WT cells.^46^ Concerning the spatial mapping of fumarate in cells, the fumarate signal (1401 cm^−1^) increased from nucleus to mitochondria for the Fh1‐KO cells, giving a first insight into its distribution.^46^ As regards to fumarate detection in mouse kidney tissue (Figure 5), the authors examined tissues from an inducible transgenic mouse model of Fh1‐loss (two *Fh1^fl/fl^*, two with induced FH loss: *Fh1^−/−^*).^46^ Raman spectroscopy measurements were performed in a blinded manner and tissues were assigned to the *Fh1^fl/fl^* or *Fh1^−/−^* groups based solely on the Raman data; their experimental status was confirmed only after these assignments were carried out.^46^ Raman imaging revealed clear spatial heterogeneity in fumarate distribution across kidney sections; more specifically, Raman images showed a clear distinction between fumarate intensities in different sections, with fumarate concentrations up to 40 mM detected in tissues that were subsequently unblinded as Fh1^−/−^.^46^ The 1401 cm^−1^ peak was diagnostic of loss of Fh1, showing similar concentrations as those found in vitro.^46^ Even though spontaneous Raman spectroscopy offers limited sensitivity thus restricting the detection of lower fumarate levels, the authors managed to demonstrate competently that the technique is very promising for the spatial mapping of metabolites in tissues.^46^

The following section highlights key challenges and general future directions that, in our assessment, are significant for advancing clinical diagnostics and biomedical research.

## 5 CHALLENGES AND GENERAL FUTURE DIRECTIONS

Undeniably, biomolecules can serve as natural labels for microscopy by measuring their molecular vibration spectra in cells and tissues.^47^ The complexity of a biological system (understood here as one of its notable aspects) is reflected by the dynamic microchemical environment found within cells and tissues, and imaging technology has advanced to the point where specific biochemicals can be targeted and visualised.^48^ Chemical imaging modalities, such as Raman imaging spectroscopy, can complement or even supplant conventional methods for profiling the biochemical makeup of tissues.^48^

As shown in the previous section, Raman scattering‐based techniques are powerful because they enable the label‐free detection of biomolecules as well as the identification of subtle biochemical changes, which makes them valuable tools for biomedical research and clinical applications. However, Raman spectroscopy faces two main challenges in the domains of clinical diagnostics and biomedical research. In recent years, there has been a clear movement towards clinically relevant studies (proof‐of‐concept).^49^ In order to achieve widespread clinical diagnostic use, standardisation is essential, for example, establishing protocols for sample preparation, instrument calibration, data acquisition and analysis to ensure reproducibility and minimise variability across clinical settings. This also includes validated reference databases for spectral interpretation and consensus guidelines for reliable disease detection. In practical terms, automated or high‐throughput instrumentation would be best suited to clinical settings for minimising pressure on personnel resource.^49^ Standardisation remains a significant challenge due to the sensitivity of Raman spectra to variations in measurement conditions and instrumental configurations.^50^ Efforts to standardise across sites typically involve spectrometer calibration and robust pre‐processing, but these methods rarely eliminate all setup‐induced differences.^50^ As demonstrated in a large‐scale cross‐laboratory study involving 35 setups across 15 institutes, even after calibration, spectral variations such as peak shifts, intensity differences and changes in peak width and noise level persist.^50^ This hampers the ability to compare data and transfer diagnostic models between sites.^50^ While computational approaches such as bilinear modelling and model transfer methods have been developed to address cross‐setup variations, a long‐term solution requires coordinated cross‐laboratory efforts and possibly device certification for clinical use.^50^ The authors of this study provided recommendations to improve inter‐laboratory comparability (e.g., robust spectrometer calibration with standard materials, unified and transparent pre‐processing steps, sharing of raw data and metadata, etc.) but also emphasised that standardisation is not yet fully resolved and is an ongoing area of work.^50^ Pertinent to biomedical research, a key limitation of current Raman studies is their fragmentation across individual research groups using different protocols, wavelengths and equipment, preventing systematic cross‐validation.^16^ Large‐scale, multi‐institutional studies and inter‐laboratory benchmarking initiatives are required to assess variability across instruments, protocols and methods^16^ in order to ensure the robustness of the discovered Raman‐based biomarkers. Another limitation that hinders research efforts is the lack of comprehensive publicly available reference spectral databases for different tissue components across various disease states to facilitate cross‐study comparisons. To standardise protocols and practices, and generate meaningful biomedical insights, it is essential to promote interdisciplinary partnerships among spectroscopists, biologists, biochemists, bioinformaticians, computational scientists and clinicians in a sustained and collaborative manner.

Moving forward with confocal Raman microspectroscopy as a label‐free modality for tissue characterisation, disease diagnosis and therapeutic monitoring, the analysis of biomedically relevant spectral band assignments and the identification of biomarkers derived from Raman spectra would result in the formation of more explicit connections between spectral feature variations and the underlying biomolecular or pathological changes in tissues. For example, comparing Raman spectral profiles across different cancer subtypes can identify unique vibrational biomarkers linked to differences in nucleic acids, proteins, lipids or metabolites, which are characteristic of each subtype; these molecular fingerprints can serve as diagnostic indicators and may also inform the development of personalised treatment strategies. Furthermore, the technique allows for capturing the effects of cancer progression and treatment response over time by tracking the temporal variations of specific Raman spectral signatures. This time resolved spectral data can reveal how tumours evolve and respond to therapy, consequently enhancing the ability to monitor pathological changes and tailor interventions more precisely. Broadly, the spatial mapping and temporal tracking of dynamic biochemical changes, as well as the assessment of drug effects and treatment responses in tissue‐engineered constructs (e.g., organoids) and patient‐derived tissue can aid in the investigation of disease progression mechanisms. Similarly, other diseases (e.g., neurodegenerative, cardiovascular, infectious) can benefit from the spatiotemporal spectroscopic tracking of tissue‐level biochemical changes.

From a computational standpoint, statistical^51^ and machine learning^52^ methods will continue to play key roles in discerning subtle spectral differences associated with various disease states or treatment responses. Robust pre‐processing pipelines can enhance signal‐to‐noise ratios in complex biological samples. Refined statistical and machine/deep learning methods can better resolve overlapping spectral signatures in complex tissues, but also can optimise spectral classification models, for example, disease staging and grading. Explainable artificial intelligence techniques can interpret spectral biomarkers and enhance clinical trust,^53^ while open‐source software tools for Raman data analysis can facilitate collaboration and reproducibility.^54^ Specifically, the repurposing of existing bioinformatics tools for Raman spectral data analysis offers a readily accessible computational framework, therefore reducing the developmental overhead typically associated with building custom pipelines from scratch.

From a biomedical perspective (clinical translation as well as mechanistic and therapeutic studies), multimodal biomarker discovery can be optimised by combining Raman spectroscopy with other imaging and omics technologies. For example: (i) combining Raman and MALDI imaging can improve spatial resolution and metabolite identification, (ii) combining Raman with fluorescence microscopy, atomic force microscopy and scanning electron microscopy (correlative microscopy) can provide enhanced functional and structural insights, and (iii) combining Raman spectromics with spatial transcriptomics, proteomics and metabolomics (spatial multiomics fusion) allows for comprehensive molecular profiling. It has been established that advances in multiplex imaging and associated computational workflows have permitted the detailed spatial and phenotypic analysis of the tumour immune microenvironment.^55^, ^56^ In a similar fashion, spatially resolved Raman imaging can facilitate the biomolecular study of tissue heterogeneity and microenvironmental interactions. For example, studying the exchange of metabolites between cancer cells and stromal/immune cells in the tumour microenvironment can provide insights into how these interactions drive tumour growth, immune evasion and resistance to treatment. In this context, assessing the way therapeutic interventions alter metabolite distribution and compartmentalisation can also provide information into mechanisms of action. In addition, complementary cell‐level advances show how vibrational readouts can link to molecular profiles and metabolism. Kobayashi‐Kirschvink et al. predicted single‐cell RNA sequencing profiles from Raman images using either anchor‐based integration with single molecule fluorescence in situ hybridisation, or anchor‐free generation with adversarial autoencoders, inferring the expression profiles of various cell states.^57^ Valera et al. utilised SERS to profile secreted purines in methylthioadenosine phosphorylase‐deficient tumours (corroborated by liquid chromatography‐mass spectrometry measurements and RNA sequencing) revealing a unique paracrine crosstalk in the tumour microenvironment.^58^ Both studies did not analyse tissue sections, but are cited as exemplars of utilising Raman scattering for translating Raman images into single‐cell expression profiles and monitoring metabolites within cell environments.

## 6 CONCLUSIONS

Raman spectroscopy has shown great potential by facilitating the detailed and label‐free analysis of biological samples with minimal to no sample preparation. This work focused on confocal Raman microspectroscopy for spatially resolved biochemical tissue characterisation. If some of the challenges are addressed, the technique could make a significant impact in biomedical research and the routine clinical setting. This may lead to early disease detection and perhaps a deeper understanding of diseases. The key to unlock its full potential will depend on interdisciplinary collaboration, thorough validation, and the development of accessible methods for analysis.

## AUTHOR CONTRIBUTIONS

*Conceptualisation and writing—original draft*: Nektarios A. Valous. *Writing—review and editing*: Nektarios A. Valous, Inka Zörnig and Dirk Jäger. All the authors read and approved the final manuscript.

## CONFLICT OF INTEREST STATEMENT

The authors declare they have no conflicts of interest.

## FUNDING INFORMATION

The authors received no specific funding for this work.

## ETHICS STATEMENT

Not applicable.

## Data Availability

Data sharing not applicable to this article as no datasets were generated or analysed during the current study.
